# The Role of Herbal Medicine in the Treatment of Acne Vulgaris: A Systematic Review of Clinical Trials

**DOI:** 10.1155/2022/2011945

**Published:** 2022-06-15

**Authors:** Ana Carolina Proença, Ângelo Luís, Ana Paula Duarte

**Affiliations:** ^1^Health Sciences Research Centre (CICS-UBI), University of Beira Interior, Avenida Infante D. Henrique, Covilhã 6200-506, Portugal; ^2^Pharmaco-Toxicology Laboratory, UBIMedical, University of Beira Interior, Estrada Municipal 506, Covilhã 6200-284, Portugal

## Abstract

Over the past few decades, interest in medicinal plants and phytochemicals for the treatment of skin disorders, including acne vulgaris, has progressively increased. Acne vulgaris is a chronic inflammatory disease of the pilosebaceous unit, which mainly occurs in adolescents and young adults. The treatment focuses on the four main factors involved in its pathogenesis: increased sebum production, hyperkeratinization, overgrowth of *Cutibacterium acnes*, and inflammation. The treatment includes topical retinoids, benzoyl peroxide, antibiotics, and oral isotretinoin. In this regard, the use of herbal medicine as a complementary and alternative medicine is a promising strategy. The main objective of this study was to systematically evaluate the efficacy and safety of medicinal plants and phytochemicals in the treatment of acne vulgaris. Three scientific databases (PubMed, Web of Science, and Scopus) were searched from inception to January 2021. Clinical trials comparing herbal therapies with placebo or other medicines for the treatment of acne vulgaris were included and analyzed. Outcome measures of interest comprised acne lesions (inflammatory and noninflammatory), sebum production, acne severity, and quality of life. The risk of bias in the included randomized controlled trials (RCTs) was assessed using the Cochrane risk-of-bias tool. A total of 34 clinical trials involving 1753 participants met the inclusion criteria for this systematic review. Most trials showed that herbal medicine significantly reduces inflammatory and noninflammatory acne lesions and has a relevant effect on acne severity. Some medicinal plants revealed equal or higher efficacy to standard treatments. No significant difference between groups in sebum production and quality of life was observed and no severe adverse events were reported. This systematic review provides evidence that medicinal plants and phytochemicals are promising treatments for mild to moderate acne vulgaris. However, more quality of evidence and standardized methodologies are needed to support their effectiveness and safety claims.

## 1. Introduction

Acne vulgaris, one of the most common dermatological conditions, is a chronic inflammatory disease of the pilosebaceous unit, affecting more than 85% of adolescents and young adults, particularly males [1–3]. Although uncommon in adulthood, recent epidemiological data point to an increasing prevalence, around 40%, predominantly in females [3–6]. The main clinical manifestations of acne are noninflammatory and inflammatory lesions, which occur primarily on the face, neck, trunk, and back [7]. Acne is generally a mild and self-limiting condition, but in its most severe form it can result in scarring and hyperpigmentation of the skin. Sequelae have a strong impact on the quality of life of individuals and are often associated with the development of psychiatric disorders [8–11].

The pathogenesis of acne is a multifactorial process that involves four main pathophysiological factors: hyperplasia and hyperproduction of sebaceous; hyperkeratinization of the sebaceous ducts; bacterial colonization and proliferation, mainly by *Cutibacterium acnes*; and inflammatory response [12, 13].

The hormonal changes typical of puberty, particularly, the increase in androgen levels, are considered the main triggers of the pathology [14, 15]. In the sebaceous glands, the type I 5 *α*-reductase enzyme reduces androgens to dihydrotestosterone, a more potent androgen, which stimulates lipogenesis and the proliferation and differentiation of sebocytes [12, 13, 16]. With increased sebum production, linoleic acid levels decrease [17], being the deficit of this compound in sebum responsible for the penetration of free fatty acids, synthesized from triglycerides, in the follicular barrier. In the follicle, fatty acids induce the production of several cytokines, such as interleukins IL-8 and IL-1*α*, involved in inflammation and keratinocyte proliferation [12, 16, 17]. In parallel, androgens promote the abnormal multiplication and differentiation of intrafollicular keratinocytes, which results in hyperkeratinization of the sebaceous duct [18, 19].

The gradual concentration of sebum and cells within the sebaceous duct leads to the development of the microcomedone, the microscopic precursor of all acne lesions, which transitions into a clinically visible lesion, i.e., an open or closed comedone. Subsequently, colonization of the follicle by *C. acnes* and the release of inflammatory mediators in the surrounding dermis encourage progression to an inflammatory lesion (papule, pustule, nodule, or cyst) [13, 19].

*C. acnes* is a Gram-positive anaerobic commensal bacterium that, through several mechanisms, stimulates the inflammatory and immune responses [13, 20]. The virulence factors secreted by this bacterium include lipases, responsible for the hydrolysis of triglycerides present in sebum; proteases and hyaluronidases, which damage the dermal and epidermal extracellular matrix; and porphyrins, molecules capable of generating reactive oxygen species and stimulating the production of IL-8 and prostaglandin PGE2 by keratinocytes [16, 21–23]. Additionally, *C. acnes* interacts with markers of the innate immune system, particularly with Toll-like receptors expressed by monocytes and keratinocytes that, once activated, secrete proinflammatory cytokines that recruit neutrophils to the pilosebaceous unit [20, 22–24]. Some recent studies have shown that *C. acnes* may reside in the pilosebaceous follicle in macrocolonies or biofilms and that these are directly related to the bacteria's resistance to antibiotics [23, 25].

According to the European guidelines, the treatment of acne vulgaris is based on the type and severity of acne, considering the patient's comorbidities and preferences [26, 27]. For mild to moderate comedogenic acne, the administration of topical agents is recommended, particularly retinoids, benzoyl peroxide, and azelaic acid [26]. Topical monotherapy treatment is usually sufficient to control the symptoms of mild acne [28]. For mild to moderate papulopustular acne, the administration of fixed combinations of benzoyl peroxide with adapalene or benzoyl peroxide with clindamycin is strongly recommended. In more severe cases, topical retinoids, namely, adapalene, can be associated with systemic antibiotics [26]. For severe papulopustular acne or moderate to severe nodular acne, treatment with oral isotretinoin monotherapy is recommended. In women, the administration of antiandrogenic hormonal therapy associated with systemic antibiotics and/or topical treatments other than antibiotics can also be considered [26].

Topical treatment includes retinoids (adapalene, tretinoin, and isotretinoin), benzoyl peroxide, azelaic acid, and antibiotics (erythromycin and clindamycin) [26]. Retinoids suppress comedogenesis, reduce sebum production, and normalize epithelial desquamation, in addition to having anti-inflammatory activity [27, 29]. Benzoyl peroxide has antibacterial and anti-inflammatory activities and exhibits mild comedolytic activity. Similarly, azelaic acid has antimicrobial, anti-inflammatory, and comedolytic properties and does not give rise to bacterial resistance [28]. Topical antibiotics have antibacterial and anti-inflammatory action, but they are not recommended in monotherapy, due to the potential development of bacterial resistance, and should be combined with benzoyl peroxide [26, 28].

Systemic treatment includes oral antibiotics, oral isotretinoin, and hormone therapy. The most used oral antibiotics are tetracyclines (doxycycline, minocycline, and lymecycline) and macrolides (erythromycin, clindamycin, and azithromycin) [27, 29]. Isotretinoin is the only drug that acts on the four pathological factors of acne, making it the most effective treatment available. It is usually reserved for cases of severe acne; however, it can be used for cases of moderate acne that do not respond to conventional therapy [29]. Finally, hormonal therapy is recommended in women with persistent inflammatory acne that is refractory to conventional treatment, with severe seborrhea, and with late-onset acne [29]. Hormonal agents include androgen receptor inhibitors (cyproterone acetate and spironolactone) and inhibitors of androgen production by the ovaries (oral contraceptives) and adrenal glands (glucocorticoids) [6, 29].

Although several therapeutic options are available for the treatment of acne, potential adverse effects, inadequate response to therapy, and the high costs associated with some treatments encourage an increased demand for alternative and complementary therapies, particularly of natural origin [30, 31]. For example, isotretinoin and its commercially available brands, although effective in the treatment of acne, can cause developmental abnormalities in the fetus (teratogenic effects) and therefore should not be used during pregnancy due to the risk of birth defects. The range and severity of associated abnormalities vary [30, 31]. Over the last few decades, there has been a growing interest in the use of medicinal plants as an alternative or adjuvant therapy in the treatment of acne vulgaris. This interest resulted from the need to minimize the increase in bacterial resistance to existing antimicrobials, eliminate or attenuate the potential adverse effects of conventional therapies, encourage adherence to therapy, and address inadequate responses to treatment [31].

Several studies have recently emerged on the use of medicinal plants and phytochemicals in the treatment of acne vulgaris, which motivated this systematic review of clinical trials. Thus, this study focused on reviewing the available studies on herbal medicine with a potential antiacne effect.

## 2. Methods

### 2.1. Search Strategy and Inclusion and Exclusion Criteria

Three electronic databases (PubMed, Web of Science, and Scopus) were searched from inception to January 2021. The PubMed search strategy served as a reference for the development of the search strategies for the other databases. The search terms used included the MeSH term “acne vulgaris” combined with the MeSH terms “phytotherapy,” “plants, medicinal,” “plant extracts,” and “herbal medicine” using boolean operator tools (Table 1). Studies were included if they were clinical trials evaluating the effectiveness of herbal therapies. The selected studies comprised one or more of the following outcome measures: number of acne lesions (inflammatory and noninflammatory), sebum production, acne severity, and quality of life. Two filters were used that limited the search to articles written in English and that involved humans. All studies in which the participants used oral, cutaneous, or mechanical therapies (extrinsic to the study) for the treatment of acne vulgaris during the study were excluded; studies whose therapeutic composition was not described or did not contain herbal or phytochemical products, studies where the participants had other pathologies or dermatological conditions that could interfere with the treatment or with the evaluation of the results, and studies carried out in animals were also excluded.

### 2.2. Study Selection

Following the PRISMA (Preferred Reporting Items for Systematic Reviews and Meta-Analyses) recommendations [32–34], two reviewers independently screened all titles and abstracts based on the defined inclusion criteria. Subsequently, the full text of each potentially eligible article was obtained and screened to support its inclusion in this systematic review. Any disagreement about study eligibility was solved through discussion.

### 2.3. Data Extraction and Synthesis

According to the PRISMA methodology [32–34], two authors independently reviewed and extracted the data using a prespecified protocol. In cases of discordance, a third reviewer was consulted to analyze discrepancies in data extraction. The data extracted from each study were synthesized and included the identification of the authors, publication year, study design and duration, study population (number of participants and classification of acne), details of the intervention (herbal medicine, pharmaceutical form(s), dose/frequency, and route(s) of administration), controls, outcome measures, and adverse effects.

### 2.4. Assessment of Risk of Bias

Two independent reviewers assessed the risk of bias of the included randomized controlled trials (RCTs) using the “Cochrane Guide for Review Authors on Assessing Study Quality” which is based on the “Cochrane Collaboration tool for assessing the risk of bias” [35]. The studies were classified as “low risk,” “unclear risk,” or “high risk” of bias regarding the following criteria: random sequence generation, allocation concealment, blinding (participants and personnel), blinding (outcome assessment), incomplete outcome data, selective reporting, and other sources of bias [36]. The results of the risk of bias assessment were presented in a risk of bias summary (review author's judgments about each risk of bias item for each included study), which were sketched using Review Manager 5.3 (Version 5.3.5).

## 3. Results

### 3.1. Included Studies

**Figure 1:**
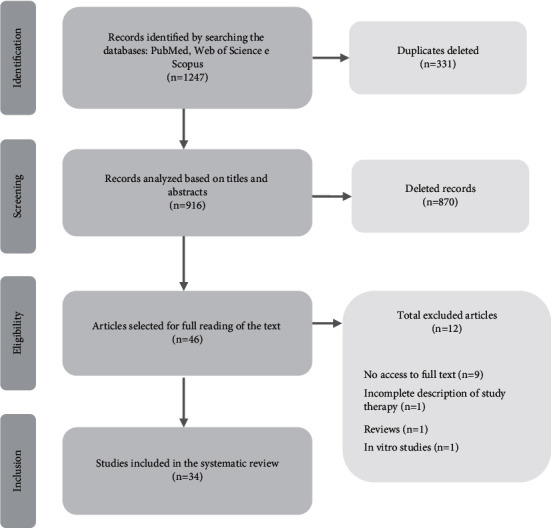
Flow diagram of the database search, trial selection, and articles included in this systematic review.

The searches in the three databases were carried out until January 2021, with a total of 1247 records having been identified. After removing 331 duplicates, 916 records were analyzed by reading the titles and abstracts, of which 46 were selected for full reading of the text, based on the inclusion and exclusion criteria. Of the 46 studies, 9 were not included in this systematic review as it was not possible to access their full texts. Other 3 studies were also excluded due to their characteristics incompatible with the defined inclusion criteria. In total, 34 studies were included in this systematic review (Figure 1).

### 3.2. Characteristics of the Studies

The characteristics of the 34 studies included in this systematic review are summarized in Table 2. Through the selection process, 34 studies were obtained, of which 25 were RCTs and 9 were non-RCTs, in which 3 were controlled and 6 were noncontrolled trials. Regarding the controlled trials, 16 compared the intervention with placebo, 6 with another approved therapy for the treatment of acne vulgaris, one with another herbal therapy, and 5 used more than one control. The duration of the studies ranged from a minimum period of 21 days to a maximum of 6 months. The studies involved a total of 1753 participants.

In 24 studies, the degree of acne severity was used as an inclusion criterion. The participants' acne was classified according to the degree of severity as follows: mild, mild to moderate, mild to severe, moderate, moderate to severe, and severe. The classification systems used were quite different between studies; however, the lesion count was the most applied classification method.

Regarding the intervention, of the 34 studies, 22 investigated a single herbal medicine, 9 tested different combinations of herbal medicines, and 3 evaluated the potential of phytochemicals in the treatment of acne vulgaris. Concerning the administration routes, the cutaneous one was the most used, followed by the oral route, and by the association of the cutaneous with the oral routes. The included studies presented several outcomes, which were used in this systematic review, namely, the number of skin lesions, the time needed to reduce 50% of the number of injuries, the area occupied by the lesions, the production of sebum, the severity of the acne, the production of porphyrins, the global clinical assessment, the evaluation by the participants, and the quality of life of the participants. Finally, 26 out of 34 studies reported the occurrence or absence of adverse effects during the study.

### 3.3. Risk of Publication Bias

**Figure 2:**
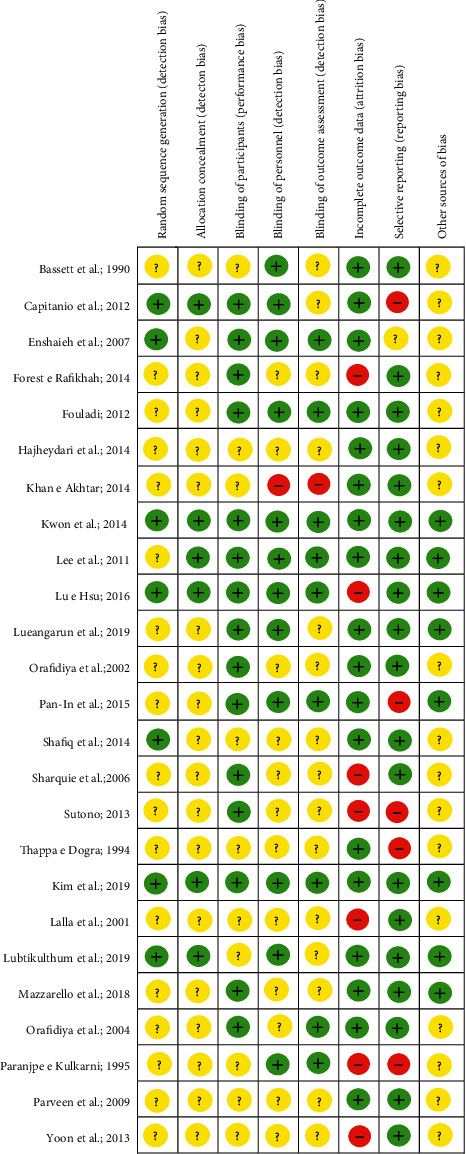
Results of risk of bias assessment regarding the methodological quality of included studies—the risk of bias summary. Review the author's judgments about each risk of bias item for each included study.

The results found in the assessment of the risk of publication bias in the 25 included RCTs are summarized in Figure 2.

In general, the included RCTs satisfied all the domains of bias defined by the Cochrane collaboration tool. Concerning the selection, performance, and detection bias, related to the allocation concealment, blinding of participants and personnel, and blinding of outcome assessment, respectively, there were several studies classified as “unclear risk,” since there were doubts regarding the allocation of participants as well as about the blinding process (single or double). In addition, other sources of bias were found, which can skew the obtained results. It is important to note, however, that the assessment of the risk of publication bias is a subjective task, even when employing the Cochrane tool, because it is based on the personal judgments of the review authors.

### 3.4. Results of the Included Trials

#### 3.4.1. Inflammatory Lesions

The number of inflammatory lesions decreased relative to baseline in the intervention groups in all studies that included this outcome. However, only in 14 studies, the change was considered statistically significant. Regarding the controlled trials that comprised this outcome, 16 of the 19 studies reported that the herbal intervention was substantially more effective in reducing the number of inflammatory lesions than the respective controls. When compared to placebo, herbal products (*L. digitata*, *C. sinensis*, *B. vulgaris*, *A. vera*, *G. mangostana*, and epigallocatechin-3-gallate) significantly reduced the number of lesions. Similar results were observed in the study by Kwon et al., with the administration of *Lactobacillus*-fermented *C. obtusa* [45]. Two other studies had better results in the intervention group than in the control one, but the changes induced by the herbal medicines, *C. sinensis* and *C. mukul*, were not statistically different from those caused by placebo and tetracycline, respectively.

In the studies by Enshaieh et al., Sharquie et al., and Mazzarello et al., the inflammatory lesions, papules, and pustules were counted individually [39]. The first two studies reported considerable differences between the intervention group and the control group in the reduction of the two types of injuries [39, 55]. In the study by Mazzarello et al., although the herbal combination under study significantly reduced the number of papules and pustules in the participants, when compared to erythromycin, the difference between the two groups only reached statistical significance in reducing the number of papules [64].

In contrast to the above results, in 3 studies, the herbal intervention was less effective in reducing the number of inflammatory lesions than the control [37, 46, 63]. In the study by Lee et al., the difference between the results achieved by the group that administered the formulation containing *Rosa* extract and the results obtained in the group that applied adapalene was not statistically significant, although the reduction in the number of lesions was higher in the control group [46]. In the studies by Bassett et al. and Lubtikulthum et al., benzoyl peroxide, administered as a control in both studies, was superior to tea tree oil and the herbal combination in reducing the number of inflammatory lesions [37, 63]. However, only the first study reported that the difference between the two groups was statistically significant [37].

#### 3.4.2. Noninflammatory Lesions

The number of noninflammatory lesions was reduced from baseline in the intervention groups in all studies that used it as an outcome. Of these studies, only 12 reported that the reduction was statistically significant. Seventeen controlled trials considered this outcome, of which 13 achieved greater reductions in the number of noninflammatory lesions in the intervention group than in the control. The changes induced by the herbal medicines *L. digitata*, tea tree oil, *B. vulgaris*, *A. vera*, and epigallocatechin-3-gallate were statistically significant when compared to placebo. Similar results were observed with the administration of *Lactobacillus*-fermented *C. obtusa* when compared to tea tree oil [45]. In the studies by Forest and Rafikhah and Kim et al., the difference between the two groups did not reach statistical significance, although the reduction in the number of lesions was higher in the intervention group [40, 61]. It should be noted that in the study by Mazzarello et al., the herbal combination under study provided a greater reduction in the number of noninflammatory lesions than placebo, being this reduction lower when compared to erythromycin [64].

In 4 studies, the reduction observed in the control group was higher than the reduction achieved in the intervention group. In these studies, tea tree oil, herbal combination, formulations containing *Rosa* extract, and *C. sinensis* were compared with benzoyl peroxide, adapalene, and placebo, respectively. However, in 2 of these studies, the difference between the two groups did not reach statistical significance [46, 47].

#### 3.4.3. Total Number of Lesions

The total number of lesions, resulting from the sum of the number of inflammatory lesions with the number of noninflammatory lesions, was reduced relative to the beginning of the study in the intervention groups and in all studies that included it as an outcome. Still, only 7 studies mentioned that the change was statistically significant. In 5 of the 9 controlled trials that integrated this result, the herbal medicines tea tree oil, *C. sinensis*, *B. vulgaris*, and *A. vera* significantly reduced the total number of lesions when compared to placebo. Similar results have been reported with the administration of the propolis-tea tree oil-*A. vera* formulation compared to erythromycin [64]. In the study by Sutono, although the reduction in the total number of lesions was higher in the group that administered *G. mangostana*, the difference between the reduction achieved in this group and the reduction achieved in the group that administered placebo was not statistically significant [57].

Diverging from other results, in the studies by Lee et al., Lu and Hsu, and Lubtikulthum et al., the herbal medicines *Rosa*, *C. sinensis*, and the herbal combination were less effective than adapalene, placebo, and benzoyl peroxide, respectively, in reducing the total number of lesions [46, 47, 63]. However, the first two studies reported that the difference between the two groups was not statistically significant [46, 47].

#### 3.4.4. Time Needed to Reduce 50% the Number of Lesions

The time needed to reduce 50% the number of lesions was used as an outcome in 2 studies, which included 210 participants [51, 65]. In the first study, several formulations were administered with increasing concentrations of the *O. gratissimum* essential oil (0.5%, 1%, 2%, and 5%) dispersed in different bases (polysorbate 80, cetomacrogol, petrolatum, and alcohol) [51]. In addition to being compared to each other, the different preparations were compared with benzoyl peroxide and with placebo. It was found that the reduction in the number of pustules was faster in preparations with high concentrations (2% and 5%) of *Ocimum* oil and with bases containing cetomacrogol or alcohol in their composition. These preparations were statistically more effective than benzoyl peroxide and placebo in reducing the number of pustules (*p* < 0.05) [51].

The second study evaluated the effect of *A. vera* on the activity of the *O. gratissimum* essential oil [65]. In the preparation, *Ocimum* oil was dispersed in increasing concentrations (0%, 25%, 50%, and 100%) of *A. vera*, which were later compared with placebo, with negative control (*A. vera* gel), and with positive control (clindamycin). The results achieved with the administration of the preparations with lower concentrations (0% and 25%) of *A. vera* were similar to the results presented by the group that administered clindamycin, whereas the preparations with higher concentrations (50% and 100%) of *A. vera* gave significantly better results than the positive control (*p* < 0.05) [65]. The number of inflammatory lesions decreased by 50% or more in all participants who administered *Ocimum* oil within a period of 2 to 5 days. The group that administered the negative control (*A. vera* gel) did not show a significant reduction in inflammatory lesions when compared to the groups that administered the herbal preparations and the group that applied the placebo did not achieve a 50% reduction in the number of lesions [65].

#### 3.4.5. Occupied Area by the Lesions

Only one study, which involved 20 participants, evaluated the effect of an herbal formulation on the area occupied by the inflammatory lesions [56]. The results revealed that in the areas where the essential oil of *C. langsdorffii* was administered, there was a significant decrease (*p* < 0.01) in the extension affected by the lesions. On the other hand, in the areas where the placebo was applied, an increase in the surface occupied by the lesions was verified in several participants [56].

#### 3.4.6. Sebum Production

In total, 6 studies, involving 254 participants, investigated the action of herbal medicine in the production of sebum. In all studies, the amount of cutaneous sebum, determined using a Sebumeter®, was reduced compared to the start of the study. However, only 2 studies reported that the reduction was statistically significant (*p* < 0.05) [43, 59].

Of the 5 controlled trials that integrate this outcome, 2 achieved a statistically significant difference between the intervention and the control groups (*p* < 0.05). In these studies, the herbal medicines *H. rhamnoides*, *C. fistula*, and *Lactobacillus*-fermented *C. obtusa* were more effective in reducing sebum production than placebo and tea tree oil, respectively [44, 45]. In the remaining studies, the difference between the intervention group and the control group was not considered significant, as the decrease in sebum production was similar in both groups.

#### 3.4.7. Acne Severity

In order to assess the effectiveness of herbal medicine in the treatment of acne vulgaris, 16 studies, which included 699 participants, used the alteration of the degree of acne severity. The studies that integrated this outcome used several classification systems, based on clinical examinations and photography.

In all studies, the degree of acne severity was reduced in the intervention group, relatively to the beginning of the study. However, only 11 studies considered the change to be statistically significant. In total, 12 controlled studies included this outcome, of which 7 reported that the herbal medicines tea tree oil, *B. vulgaris*, *A. vera*, *G. mangostana*, and Unani formulation, and the phytochemicals resveratrol and epigallocatechin-3-gallate were significantly more effective than placebo in reducing in the degree of severity of acne (*p*=0.000; *p* < 0.001; *p*=0.001; *p*=0.042; *p* < 0.0001; *p* < 0.001; *p* < 0.05, respectively) [39, 41, 42, 52, 67, 68, 70].

In the study by Kwon et al., participants in the group that administered the formulation containing *Lactobacillus*-fermented *C. obtusa* considerably reduced the degree of acne severity when compared to those who administered tea tree oil (*p* < 0.05) [45]. In the study by Mazzarello et al., the group that administered the propolis-tea tree oil-*A. vera* formulation achieved a greater reduction in severity than the groups that administered placebo and erythromycin [64]. The difference between the results achieved in the intervention group and in the group that administered erythromycin was statistically significant (*p* = 0.0368) [64]. In the study by Kim et al., the *Cheongsangbangpoong-tang* formulation promoted a reduction in the severity of acne, but the results were not statistically different from those of the group that administered the placebo [61]. Similarly, in the study by Shafiq et al., the results achieved by the group that administered the herbal medicine *C. equisetifolia* were not significantly different from the results presented by the group that applied benzoyl peroxide [54].

On the other hand, in the study by Lee et al., the reduction in acne severity of the participants who administered the formulation containing the *Rosa* extract was minor than the reduction achieved with the administration of adapalene; however, the difference between the two groups was not considerable (*p* = 0.641) [46].

#### 3.4.8. Porphyrin Production

The concentration of porphyrins, which indirectly reveals the amount of *C. acnes* in the skin, was used by 3 studies as an outcome, including 165 participants. Different quantification methods were employed in the various studies, namely examination using Wood's lamp, image analysis based on UV photography, and the VISIA® analysis system.

All herbal medicines, *G. mangostana*, *M. communis*, and the combination of *A. cepa*, *Lavandula*, *G. mangostana*, *A. vera*, *M. papyrifera*, and *M. alternifolia* significantly reduced (*p* < 0.001; *p* < 0.0001; *p*=0.003, respectively) the concentration of porphyrins, in relation to the beginning of the study, thus demonstrating their antibacterial properties [48, 53, 63]. Additionally, the efficacy of *G. mangostana* and the herbal combination was compared with that of clindamycin and benzoyl peroxide, respectively. In both studies, the difference between the changes observed in the intervention group and in the control group was not statistically significant (*p*=0.649 and *p*=0.425) [48, 63].

#### 3.4.9. Global Clinical Evaluation

Six studies, involving 360 participants, described the overall response to treatment as an outcome. The response to treatment was assessed by specialists who were guided by scales defined by each of the studies.

In the study by Khan and Akhtar, the response to treatment with the herbal medicines, *H. rhamnoides* and *C. fistula,* was classified as “excellent,” “good,” or “undefined,” relative to the beginning of the study. At the end of the study, of the 31 participants with Grade I (mild) acne, 9 had an “excellent” response, and 17 had a “good” response to treatment. As for the 19 participants with Grade II (moderate) acne, 4 responded “excellent” to the treatment, and 13 responded “good” [44]. Similarly, in the study by Shafiq et al., the response to treatment with *C. equisetifolia* was also categorized [54]. The study results demonstrated that the number of participants who achieved a response rated “excellent” or “good” was higher in the intervention group than in the benzoyl peroxide group [54]. Additionally, in the study by Lalla et al., the response to treatment was rated from “excellent” to “poor”. Several conclusions were drawn from the results of this study: (1) the two groups of participants who administered the ayurvedic formulation, orally and dermally, had a higher number of excellent responses to treatment than the group of participants who administered the ayurvedic formulation orally only; (2) of the two groups that administered the ayurvedic formulation orally and topically, the group that administered the cream formulation had a higher number of excellent responses than the group that administered the gel formulation (57.89% vs 31.58%); (3) the control group that simultaneously administered placebo preparations orally and topically did not obtain any response [62]. In the study by Paranjpe and Kulkarni, only one of the Ayurvedic formulations, called Sunder Vati, gave rise to significant changes in relation to the beginning of the study. Approximately two-thirds of participants who administered this formulation exhibited a “good” to “excellent” clinical response at the end of the study [66].

According to the study by Lee et al., the formulation containing *Rosa* extract provided a considerable improvement in acne in 84% of participants, compared to the beginning of the study. However, the results did not differ significantly from the group that administered adapalene (*p* = 0.303), which generated a significant response in 97% of participants [46]. Finally, in the study by Lueangarun et al., *G. mangostana* promoted the regression of acne more markedly than clindamycin. The difference between the two groups was statistically significant (*p*=0.004) [48].

#### 3.4.10. Participants' Evaluation

In total, 5 studies, which included 280 participants, used the opinion of individuals as a method of evaluating the effectiveness of treatment. Thus, in the studies by Lee et al. and Malhi et al., the participants evaluated the evolution of acne during treatment [46, 49]. In the first study, 77% of participants treated with a formulation containing *Rosa* extract said that their acne significantly improved compared to the baseline, but the results were not statistically different from those reported by participants who administered adapalene (*p*=0.314) [46]. In the second study, at the end of each week of tea tree oil treatment, participants looked at whether the severity of acne had changed from the previous week. The most frequent answers were that the acne was the same (46%) or slightly better (43%) [49].

The remaining studies assessed participants' satisfaction with the treatment. In the study by Lueangarun et al. (2019), the participants showed high satisfaction (*p* < 0.001) with the administration of the formulation containing *G. mangostana*, as well as with the administration of the clindamycin gel, with no statistically significant difference being reached between the two treatments (*p*=0.714) [48]. Regarding the study by Sharquie et al., the participants who administered the herbal medicine *C. sinensis* revealed levels of satisfaction higher than those who used placebo [55]. Finally, in the study by Lubtikulthum et al., the satisfaction with the treatment efficacy was similar in both groups (*p*=0.391); however, the participants expressed greater satisfaction with the administration of the herbal combination than with the administration of benzoyl peroxide, which resulted in a difference statistically significant (*p*=0.011) [63].

#### 3.4.11. Participants' Quality of Life

Three studies, with a total of 191 participants, evaluated the impact of herbal treatment on the participants' quality of life. In the study by Lu and Hsu, the quality of life of the participants, determined using the Cardiff Acne Disability Index (CADI) questionnaire, did not vary significantly in relation to the beginning of the study (*p*=0.28). Furthermore, the difference between the results obtained with the herbal medicine *C. sinensis* and with the placebo did not reach statistical significance (*p*=0.83) [47].

In the remaining studies, the herbal *A. lappa* and the combination of extracts from *A. cepa*, *Lavandula*, *G. mangostana, A. vera*, *M. papyrifera*, and *M. alternifolia* promoted a significant improvement (*p* < 0.001) in the quality of life of the participants, regarding the beginning of the study, according to the questionnaires used [50, 63]. The results obtained with the administration of the herbal combination were also compared with those of benzoyl peroxide, but there were no statistically significant differences between the two groups (*p*=0.344) [63].

## 4. Discussion

This systematic review included 34 studies with a total of 1753 participants, which evaluated the efficacy of herbal medicine in the treatment of acne vulgaris. The evidence presented by the studies suggests that herbal and phytochemical formulations can be effective in the treatment of acne vulgaris, as demonstrated by the reduction in the number of lesions, the production of sebum, the severity of the pathology, and the production of porphyrins, as well as for the improvement of the participants' quality of life, observed in the intervention group in several studies. In most controlled trials, the intervention group achieved results equal to or better than the control group, with some studies showing that the difference between groups was statistically significant.

The different therapeutic strategies employed showed the versatility with which herbal products can be introduced in the daily treatment of acne vulgaris. Monotherapy was the most used strategy, followed by the association of herbal medicine with standard acne treatments. This last strategy, called adjuvant therapy, proved to be promising as it allowed to reduce the initial dose of certain drugs and, therefore, the adverse effects associated with their administration. Additionally, several studies have reported synergistic therapeutic effects when different herbal medicines were combined.

Considering the results of the studies and the quality of the evidence presented, the botanical species *Melaleuca alternifolia*, *Camellia sinensis*, *Berberis vulgaris*, and *Chamaecyparis obtusa* fermented by *Lactobacillus*, *Garcinia mangostana*, and *Aloe vera*, were the most employed in the included clinical trials.

Concerning some adverse effects that are reported in clinical trials included in this systematic review, the overall results of the studies employing tea tree oil revealed that it is as effective as benzoyl peroxide in reducing inflammatory lesions, but benzoyl peroxide has a faster onset of action [37, 39, 49]. Still, subjects who administered tea tree oil experienced fewer adverse effects (dryness, itching, burning, and flushing) than those who administered benzoyl peroxide [37]. Following these results, tea tree oil presents itself as an alternative therapy to conventional treatments of mild to moderate acne vulgaris, acting simultaneously as an antibacterial and anti-inflammatory. Given its broad-spectrum antibacterial activity, tea tree oil may be a viable option in the treatment of therapy-resistant acne. The minimal adverse effects associated with its administration and the absence of teratogenicity encourage its use in the treatment of acne vulgaris [39].

Tea tree oil is an essential oil extracted from the plant native to Australia, *Melaleuca alternifolia* [71]. Considered as a medicinal essential oil, it has been used for several decades in the treatment of skin disorders [49, 72]. Consisting of more than 100 components, tea tree oil has terpinen-4-ol as its major compound, which corresponds to at least 35% of the oil [71, 73]. Terpinen-4-ol has strong antimicrobial and anti-inflammatory activity and properties that support the use of tea tree oil in the treatment of acne vulgaris [71, 74, 75]. The antimicrobial mechanism of action of this oil involves structural and functional changes in the bacterial membrane [75]. Several studies investigated the antimicrobial activity of the essential oil on *C. acnes*, having reported that the minimum inhibitory concentration (MIC) of the oil for the bacterium is between 0.3 and 0.6% and the minimum bactericidal concentration (MBC) is between 0.25 and 0.5% [45,76–78]. The second property of tea tree oil that contributes to its therapeutic efficacy is its anti-inflammatory activity. *In vitro*, the main constituent of the oil reduced the production of inflammatory mediators, such as TNF-*α*, IL-1*β*, IL-8, IL-10, and prostaglandin (PG) E2 [79]. Additionally, the water-soluble fractions of tea tree oil, terpinene-4-ol, and *α*-terpineol suppressed monocyte superoxide production [80].

*Camellia sinensis* is a plant native to Southeast Asia, from which the second most consumed beverage in the world, tea, is produced [81, 82]. From its leaves, four varieties of tea are derived, white tea, green tea, oolong tea, and black tea, whose composition varies according to the fermentation and drying processes to which the leaves are subjected [83]. White tea and green tea are not fermented, differing in the maturity of the leaf used in their production, oolong tea is partially fermented, and black tea is fully fermented [83, 84]. The fermentation process generates conformational changes in the bioactive components of tea, which results in changes in its biological properties [83, 85, 86]. Green tea is made from fresh leaves of *C. sinensis* processed to prevent oxidation of its polyphenolic compounds [87, 88]. Catechins are the main polyphenols present in green tea, representing about 30% to 42% of the water-soluble solids of this tea [89]. Its content is influenced by several factors, such as geographic location, growing conditions, and the degree of fermentation [83]. The four main catechins present in tea are epigallocatechin-gallate, epicatechin-gallate, epigallocatechin, and epicatechin [90]. Epigallocatechin-gallate is the most abundant catechin in green tea, accounting for about 59% of the total catechins, and the most important from a pharmacological point of view [91–93]. Numerous pharmacological properties have been attributed to green tea, highlighting the antioxidant, anti-inflammatory, antimicrobial, and anticancer properties [91, 94]. The antioxidant activity of green tea, mediated by catechins, occurs through the induction of antioxidant enzymes, the scavenging of free radicals, and the inhibition of lipid peroxidation [86]. This property is considered the most important of this class of polyphenols since its anti-inflammatory action derives from its action as an antioxidant [95–97]. On the other hand, its antimicrobial activity results from alterations in the bacterial membrane and from the inhibition of fatty acid synthesis and the enzymatic activity of bacteria [91, 98]. In addition to these activities, recent studies suggest that green tea reduces sebum production by inhibiting the 5*α*-reductase enzyme [92, 99]. Given these properties, green tea acts directly on three of the four pathological mechanisms involved in the pathogenesis of acne vulgaris. From the 3 studies included in this review that investigated the efficacy of green tea in the treatment of acne vulgaris, it is possible to conclude the following: green tea considerably reduces inflammatory lesions, but does not exert significant effects on noninflammatory lesions; green tea is more effective in treating mild to moderate acne than moderate to severe acne; oral administration of green tea is as efficient as cutaneous administration; few adverse effects are associated with the administration of green tea. Following this evidence, it is possible to state that green tea could be an alternative to conventional treatments for mild to moderate acne vulgaris.

*Berberis vulgaris* is a plant of the *Berberidaceae* family widely found in Europe, Asia, and America [100]. The reddish fruit of this plant is commonly included in gastronomic dishes, while the roots, stems, and bark are used in traditional medicine [101]. The medicinal properties of *B. vulgaris* are mostly attributed to berberine, an isoquinoline alkaloid that belongs to the structural class of protoberberines [102]. Berberine exhibits multiple pharmacological properties, including anti-inflammatory, antioxidant, antibacterial, antifungal, and anxiolytic properties [103]. Additionally, a study has shown that berberine considerably suppresses lipogenesis in the sebaceous glands [104]. The potential beneficial effects of *B. vulgaris* motivated the investigation of its therapeutic efficacy in the treatment of acne vulgaris. The effects of aqueous extract of the *B. vulgaris* fruit on adolescents with moderate to severe acne vulgaris were evaluated. After 4 weeks of treatment, the number of inflammatory and noninflammatory lesions, as well as acne severity were significantly reduced, with no adverse effects or associated complications. The evidence suggests that the success of the treatment resulted from the anti-inflammatory action, exerted mainly by the alkaloid fraction of *B. vulgaris*, from the antioxidant action, through the elimination of free radicals and the inhibition of lipid peroxidation, and from the anxiolytic action, since acne exacerbations are often related to bouts of anxiety and stress [105].

*Chamaecyparis obtusa* is a species of cypress native to Asia, which has been widely used as a cosmetic, perfume, and disinfectant [45, 106]. The essential oil extracted from its leaves contains numerous terpenes, molecules characterized by their antioxidant and anti-inflammatory properties, and specific compounds, such as *β*-tuiaplicin, which confer antimicrobial activity [45, 93, 106–113]. Recently, a study revealed that fermentation of *C. obtusa* by *Lactobacillus* substantially increases its antimicrobial activity, particularly against *C. acnes*, because of the increased content of dihydroxybenzoic acid, taxifolin, and quercetin [45]. Given the promising properties of this plant, Kwon et al. investigated the effect of *Lactobacillus*-fermented *Chamaecyparis obtusa* in the treatment of mild to moderate acne vulgaris and subsequently compared its efficacy with that of tea tree oil [45]. This study stands out for being the first clinical trial, to date, to compare the efficacy and safety of two herbal medicines in the treatment of acne vulgaris. The results of this study showed that the two herbal medicines were effective in reducing the number of inflammatory and noninflammatory lesions; however, *Lactobacillus*-fermented *Chamaecyparis obtusa* was significantly superior to tea tree oil. After one week of treatment with *Lactobacillus*-fermented *Chamaecyparis obtusa*, the number of inflammatory lesions in the participants decreased considerably, indicating that *Lactobacillus*-fermented *Chamaecyparis obtusa* has a therapeutic efficacy comparable to that of topical retinoids and antibiotics, with the advantage of not having adverse effects. In contrast, tea tree oil only achieved significant reductions after four weeks of administration. Similarly, the reduction in the number of noninflammatory lesions was faster and more pronounced on the side of the face where *Lactobacillus*-fermented *Chamaecyparis obtusa* was applied. Finally, the authors elucidated the mechanism of action underlying the observed clinical results. Among the various molecules studied, the accelerated decrease in the expression of the NF-kB protein, in the area where *Lactobacillus*-fermented *Chamaecyparis obtusa* was administered, justified the stronger and faster anti-inflammatory effect of *Lactobacillus*-fermented *Chamaecyparis obtusa* compared to tea tree oil. Furthermore, sebo-suppression resulted from the reduction of the SREBP-1 protein, one of the main regulators of lipid synthesis in the sebaceous glands [45].

Mangosteen, the fruit of the *Garcinia mangostana* tree, is known as the “queen of fruits” in Southeast Asia for its distinctive flavor and numerous health benefits [114–116]. Its bark, used for centuries in the treatment of different pathologies, is currently marketed as a food supplement all over the world [115, 117, 118]. The main phytochemicals present in *G. mangostana* are xanthones, a class of secondary metabolites with biological antioxidant, anti-inflammatory, neuroprotective, antimicrobial, and antifungal effects [114, 116]. The most abundant xanthones found in this species are *α*-mangostine and *γ*-mangostine [119]. *In vitro* studies have shown that *G. mangostana*, in particular, *α*-mangostine, exerts strong antimicrobial activity against *C. acnes* and *Staphylococcus epidermidis*, bacteria involved in the pathogenesis of acne [120–122]. This activity, associated with its anti-inflammatory action, motivated the development of clinical studies that determined the anti-acne activity of *G. mangostana in vivo*. The studies included in this review that investigated the potential of *G. mangostana* in the treatment of acne vulgaris achieved promising results. In various studies, the number of inflammatory and noninflammatory lesions, the severity of acne, and the number of porphins were drastically reduced, with few associated adverse effects. The antimicrobial, anti-inflammatory, and antioxidant properties of *G. mangostana*, reported by in vitro studies, support the results obtained by clinical trials. Scientific studies have shown that *α*-mangostine, the main xanthone present in the bark of *G. mangostana*, has potent antimicrobial activity against *C. acnes*, as evidenced by a MIC of 0.039 mg/mL [121, 123]. Furthermore, *α*-mangostine exhibits anti-inflammatory activity, through the reduction of TNF-*α* and PGE2, and antioxidant activity, which results from the inhibition of reactive oxygen species [116, 124]. Taken together, these properties validate the use of *G. mangostana* as an alternative therapy in the treatment of acne vulgaris.

*Aloe vera*, the most popular species belonging to the genus *Aloe*, is one of the most used herbal medicines worldwide for its immeasurable health benefits [125–127]. Native to the Arabian Peninsula, *A. vera* is a xerophytic plant characterized by its long green leaves, with thorny margins, filled with a mucilaginous pulp (*A. vera* gel) rich in water and bioactive components that concentrate numerous properties [125, 128]. More than 75 different components were identified in the *A. vera* gel, including polysaccharides, anthraquinones, flavonoids, terpenes, saponins, amino acids, minerals, and vitamins [129–131]. Anthraquinones are the most important secondary metabolites present in *A. vera* gel, being responsible for the astringent, antibacterial, anti-inflammatory, antioxidant, and healing properties attributed to *A. vera* [126]. These properties, which are crucial in the treatment of skin conditions, have stimulated the investigation of the antiacne activity of *A. vera* gel *in vivo*. The *A. vera* gel minimized the adverse effects associated with the administration of tretinoin, an effect attributed to its anti-inflammatory and soothing properties. Additionally, the results of the clinical trials revealed that epigallocatechin-3-gallate is effective in reducing inflammatory and noninflammatory lesions, with few adverse effects. Taken together, the evidence from the studies suggests that epigallocatechin-3-gallate may represent a new therapeutic opportunity in the treatment of acne vulgaris.

The present systematic review has some limitations. Of the included articles, only RCTs were evaluated for the risk of bias, so the evidence from the remaining studies may be subject of high risk. Another limitation is related to the multiple acne classification and outcome assessment systems used by various studies. The absence of standardized and validated systems compromised the comparison of results between studies. Furthermore, some trials were performed for the same herbal medicine. Moreover, since the composition of the extracts studied in the clinical trials and included in the present meta-analysis is often unreported in the original paper, the obtained results may be not reproducible. Additionally, most studies investigated the effect of herbal medicines on individuals with mild to moderate acne, which made it impossible to generalize the results. Finally, since the formulation of the pharmaceutical dosage form and its physicochemical characteristics play a very important role in the efficacy of any dosage form, which is even more obvious when using medicinal plants which are usually prepared from different sources, it would be of major importance that before any clinical trial, the information regarding the suitability of the pharmaceutical dosage form from the physicochemical point of view including the extraction methods and standardization of the active raw materials was obtained. Otherwise, the results of clinical trials will be very different and unreliable due to the different quality of the applied dosage forms even produced from the same herb.

## 5. Conclusions

The evidence presented by the studies described suggests that herbal and phytochemical formulations can be effective in the treatment of acne vulgaris, as demonstrated by the reduction in the number of lesions, sebum production, the severity of the pathology, and the production of porphyrins, and by the improvement in the quality of life of the participants, observed in the intervention group in several studies. In most of the controlled trials, the intervention group achieved results equal to or greater than the control group, with some studies showing that the difference between groups was statistically significant.

The different therapeutic strategies used showed the versatility with which herbal products can be introduced in the daily treatment of acne vulgaris. Monotherapy was the most used strategy, followed by the association of herbal medicines with standard acne treatments. This last strategy, known as adjuvant therapy, proved to be promising, as it allowed to reduce the initial dose of certain drugs and, therefore, the adverse effects associated with their administration. Additionally, several studies have reported synergistic therapeutic effects when different herbal medicines are combined.

## Figures and Tables

**Table 1:** Search string used for this systematic review.

*PubMed*
(1) Plant (1082807)
(2) Plant extract (188557)
(3) Tea (30664)
(4) Herbal products (7154)
(5) Natural products (637692)
(6) 1 OR 2 OR 3 OR 4 OR 5 (1547065)
(7) Phytotherapy (39254)
(8) Treatment (10562707)
(9) Remedy (10139)
(10) Natural therapy (155217)
(11) Herbal medicine (41337)
(12) 7 OR 8 OR 9 OR 10 OR 11 (10578745)
(13) Acne vulgaris (12303)
(14) Propionibacterium acnes (4976)
(15) 13 OR 14 (16271)
(16) 6 AND 12 AND 15 (732)
(17) 16 AND Humans AND English (362)

*Web of Science*
(1) Plant (4958255)
(2) Plant extract (463391)
(3) Tea (125509)
(4) Herbal products (43344)
(5) Natural products (350473)
(6) 1 OR 2 OR 3 OR 4 OR 5 (5283779)
(7) Phytotherapy (39811)
(8) Treatment (9089471)
(9) Remedy (64916)
(10) Natural therapy (206773)
(11) Herbal medicine (69546)
(12) 7 OR 8 OR 9 OR 10 OR 11 (9313410)
(13) Acne vulgaris (17168)
(14) *Propionibacterium acnes* (8498)
(15) 13 OR 14 (23700)
(16) 6 AND 12 AND 15 (569)
(17) 16 AND English (547)

*Scopus*
(1) Plant (2340371)
(2) Plant extract (271770)
(3) Tea (64489)
(4) Herbal products (16588)
(5) Natural products (191436)
(6) 1 OR 2 OR 3 OR 4 OR 5 (2547351)
(7) Phytotherapy (40878)
(8) Treatment (7537055)
(9) Remedy (60029)
(10) Natural therapy (73955)
(11) Herbal medicine (53492)
(12) 7 OR 8 OR 9 OR 10 OR 11 (7665338)
(13) Acne vulgaris (14914)
(14) *Propionibacterium acnes* (8158)
(15) 13 OR 14 (21527)
(16) 6 AND 12 AND 15 (362)
(17) 16 AND English (338)

**Table 2:** Main characteristics of the included studies in this systematic review.

Author, year	Study design, duration	Participants		Intervention	Control	Outcomes	Adverse effects
		*N* (intervention group/control group)	Acne classification (severity degree; classification system)	Herbal medicine; pharmaceutical form(s); dose/frequency; route(s) of administration			
*Plant extracts*							
Bassett et al., 1990 [37]	RCT, 3 months	61/63	Mild to moderate; leeds system	Tea tree oil 5%; gel; cutaneous	Benzoyl peroxide 5%	Number of inflammatory and noninflammatory lesions	Intervention group: 44% of the participants reported dryness, itching, burning, and redness of the skin. Control group: 79% of the participants reported the same adverse effects.
Capitanio et al., 2012 [38]	RCT, 8 weeks	30/30	Mild; leeds system	A complex of zinc and an oligosaccharide derived from the seaweed *Laminaria digitata*; cream; twice a day; cutaneous	Placebo	Number of inflammatory and noninflammatory lesions; sebum production	Absence of irritation and skin peeling.
Enshaieh et al., 2007 [39]	RCT, 45 days	30/30	Mild to moderate; injury count	Tea tree oil 5%; gel; twice a day; cutaneous	Placebo	Number of total lesions; number of inflammatory and noninflammatory lesions; acne severity (ASI)	Intervention group: itching (*N* = 3); burning (*N* = 1); desquamation (*N* = 1). Control group: itching (*N* = 2); burning (*N* = 2).
Forest and Rafikhah, 2014 [40]	RCT, 30 days	18/16	Mild to moderate; leeds system	*Camellia sinensis* (aqueous extract of green tea); capsule; 500 mg/3 times per day; oral	Placebo	Number of total lesions; number of inflammatory and noninflammatory lesions	Without adverse effects
Fouladi, 2012 [41]	RCT, 4 weeks	25/25	Moderate to severe; injury count	*Berberis vulgaris* (aqueous extract of dried fruit); capsule; 200 mg/3 times per day; oral	Placebo	Number of total lesions; number of inflammatory and noninflammatory lesions; acne severity (Michaelson's acne severity score)	Without adverse effects
Hajheydari et al., 2014 [42]	RCT, 8 weeks	30/30	Mild to moderate; GAGS	*Aloe vera* topical gel combined with tretinoin cream 0.025%; gel; twice a day; cutaneous	Placebo + tretinoin	Number of total lesions; number of inflammatory and noninflammatory lesions; acne severity (ASI)	The intervention group reported fewer adverse effects than the control group
Hou et al., 2018 [43]	Uncontrolled trial, 4 weeks	20	Mild to moderate; NR	*Panax ginseng* (hydrophobic fraction in red ginseng ethanol extract); cream; 2 twice a day; cutaneous	—	Number of inflammatory and noninflammatory lesions; sebum production	NR
Khan and Akhtar, 2014 [44]	RCT, 12 weeks	(Female 1) 25/25 (Female 2) 25/25	Moderate; leeds system	(F1) *Hippophae rhamnoide*s; (F2) *Cassia fistula*; emulsion; 500 mg twice a day; cutaneous; each powdered plant was extracted with 70% methanol solution	Placebo	Sebum production; global clinical evaluation	NR
Kwon et al., 2014 [45]	RCT, 8 weeks	34/34	Mild to moderate; modified leeds system	*Chamaecyparis obtusa* fermented by *Lactobacillus*; cream; twice a day; cutaneous	Tea tree oil	Number of inflammatory and noninflammatory lesions; sebum production; acne severity (modified leeds system)	Intervention group: mild erythema (*N* = 2); skin dryness (*N* = 2). Control group: slight skin dryness (*N* = 4); moderate erythema and desquamation (*N* = 6).
Lee et al., 2011 [46]	RCT, 12 weeks	50/47	Mild to moderate; KAGS	*Rosa* combined with hexamidine disethionate 0.05% and retinol 0.03%; once a day; cutaneous	Adapalene 0.1%	Number of total lesions; number of inflammatory and noninflammatory lesions; acne severity (KAGS); global clinical evaluation; participants evaluation (TR)	The intervention group reported fewer adverse effects than the control group. However, by the end of the study, the difference between the two groups became negligible.
Lu and Hsu, 2016 [47]	RCT, 4 weeks	40/40	Moderate to severe; IGA	*Camellia sinensis* (decaffeinated green tea extract); capsule; 500 mg/3 times per day; oral	Placebo	Number of total lesions; number of inflammatory and noninflammatory lesions; life quality (CADI)	Intervention group: constipation (*N* = 1); abdominal discomfort (*N* = 2). Control group: polydipsia (*n* = 1); insomnia (*N* = 1).
Lueangarun et al., 2019 [48]	RCT, 12 weeks	28/28	Moderate to severe; GAGS	*Garcinia mangostana* (topical mangosteen extract in nanoparticle loaded gel, containing *α*-mangostin); gel; twice a day; cutaneous	Clindamycin 1%	Number of inflammatory and noninflammatory lesions; porphyrins production; clinical global evaluation; participants evaluation (TS)	Similar adverse effects in both groups. After 4-weeks of treatment, no participant had adverse effects on both sides of the face.
Malhi et al., 2017 [49]	Uncontrolled trial, 12 weeks	18	Moderate to severe; injury count and IGA	Tea tree oil; gel; twice a day; cutaneous	—	Number of total lesions; acne severity; participants evaluation (TR)	Well tolerated treatment. Moderate desquamation (*N* = 2); moderate skin dryness (*N* = 1).
Miglani and Manchanda, 2014 [50]	Uncontrolled trial, 6 months	34	NR; GAGS	*Arctium lappa*; 4 pills/4 times per day for 7 days followed by 7 days of placebo; oral	—	Number of total lesions; number of inflammatory and noninflammatory lesions; acne severity (GAGS); life quality (Acne-QoL)	NR
Orafidiya et al., 2002 [51]	RCT, 4 weeks	112/ (1) 7 (2) 7	NR; injury count	*Ocimum gratissimum* essential oil; 0.25 cm^3^/twice a day; cutaneous	(1) Benzoyl peroxide 10% (2) Placebo	Time necessary to reduce 50% of the total number of lesions (days)	Adverse effects are minimal and tolerable
Pan-In et al., 2015 [52]	RCT, 4 weeks	10/10	NR; injury count	*Garcinia mangostana* (cellulose-based nanoparticles as nano-reservoir and *α*-mangostin, an active component isolated from the edible *Garcinia mangostana* fruit); gel; twice a day; cutaneous	Placebo	Number of inflammatory lesions; acne severity (ASI)	NR
Pécastaings et al., 2018 [53]	Controlled trial, 56 days	60	Mild to moderate; GEA	*Myrtus communis* leaf extract; cream; twice a day; cutaneous	Healthy volunteers, free of facial or dorsal acne and of any facial dermatosis	Acne severity; porphyrins production	Without adverse effects
Shafiq et al., 2014 [54]	RCT 45-days	25/25	NR; injury count	*Casuarina equisetifolia* bark extract 5% with 90% methanol; cream; twice a day; cutaneous	Benzoyl peroxide	Acne severity (Cook's Acne Grading Scale); global clinical evaluation	Intervention group: without adverse effects. Control group: 17% of participants reported skin irritation and redness.
Sharquie et al., 2006 [55]	RCT, 2 months	30/30	Mild to moderate; injury count	*Camellia sinensis*; lotion; twice a day; cutaneous	Placebo	Number of inflammatory lesions; participants evaluation (TS)	Without adverse effects
da Silva et al., 2012 [56]	Controlled clinical trials, 21 days	10/10	Mild; NR	*Copaifera langsdorffii* essential oil; gel; twice a day; cutaneous	Placebo	Area occupied by the inflammatory lesions (mm^2^)	Without adverse effects
Sutono, 2013 [57]	RCT, 3 weeks	45/41	Mild to moderate; Lehman criteria	*Garcinia mangostana* (extract of mangosteen rind); capsule; 400 mg/3 times per day; oral	Placebo	Number of total lesions; number of inflammatory and noninflammatory lesions	Without adverse effects
Thappa and Dogra, 1994 [58]	RCT, 3 months	10/10	Severe (nodulocystic); injury count	*Commiphora mukul* (gugulipid, equivalent to 25 mg guggulsterone); 1 pill twice a day; oral	Tetracycline oral (500 mg)	Number of inflammatory and noninflammatory lesions	Without adverse effects

*Combinations of plant extracts*							
Beltrami et al., 2001 [59]	Controlled clinical trials, 90 days	15/15	Mild to severe; NR	*Krameria trianda*, *Serenoa repens*, and *Centella asiatica*; cutaneous	Topical treatment + placebo (oral)	Sebum production	Intervention group: burning (resolved with continued treatment).
Lone et al., 2012 [60]	Uncontrolled trial, 45 days	25	NR; Cook's system of acne grading	Unani formulation: Irsa (*Iris florentina*), barghe neem (*Azadirachta indica* leaves), poste saras (*Acacia speciosa* bark), ghungchi safaid (*Abrus precatorious*), and *Namake Sambhar* (Lake salt) 50 grams each; 6 to 10 g/once a day; cutaneous	—	Acne severity (Cook's Acne Grading Scale)	Without adverse effects
Kim et al., 2019 [61]	RCT, 8 weeks	28/28	NR; injury count	Cheongsangbangpoong-tang formulation: *Schizonepeta tenuifolia* (0.5 g), *Coptis japonica* makino (0.5 g), *Mentha arvensis* var. *iperascens* (0.5 g), *Ponciri Fructus Immaturus* (0.5 g), *Glycyrrhiza uralensis* FISCH (0.5), *Gardenia augusta* (1.0 g), *Cnidium officinale* (1.0 g), *Scutellaria baicalensis* (1.0 g), *Forsythia koreana* (1.0 g), *Angelica dahurica* (1.0 g), *Platycodon grandiflorum* (1.0 g), *Ledebouriella seseloide*s (1.0 g), corn starch (1.2 g), lactose hydrate (2.3 g); granulated; 5 g/3 times per day; oral	Placebo	Number of inflammatory and noninflammatory lesions acne severity (KAGS; IGA)	Intervention group: Digestion discomfort (*n* = 3). There were no serious adverse effects.
Lalla et al., 2001 [62]	RCT, 4 weeks	(G1) 23 (G2) 23 (G3) 5 (G4) 2	Mild to severe; Leeds system	Ayurvedic formulation (soft extracts of *Aloe barbadensis* Miller, *Azardirachta indica* Juss, *Curcuma longa* Linn, *Hemidesmus indicus* Linn, *Terminalia chebula* Retzr, *Terminalia arjuna* Rob, and *Withania somnifera* Linn (one part of the extract approximately representing four parts of dried/fresh plant material); 2 pills/twice a day + topical preparation (gel (G1) or cream (G2))/twice a day oral and cutaneous	(G3) Placebo (topical preparation) (G4) placebo (oral and topical preparation)	Participants evaluation (TR)	Mild itching (*N* = 2); increased gastric motility (*N* = 2). Reported adverse effects decreased with continued treatment.

Lubtikulthum et al., 2019 [63]	RCT, 12 weeks	39/38	Mild to moderate; modified leeds system	*Allium cepa*, *Lavandula*, *Garcinia mangostana*, *Aloe vera*, *Morus papyrifera*, and *Melaleuca alternifolia*; gel; 1 g/twice a day; cutaneous	Benzoyl peroxide 2.5%	Number of total lesions; number of inflammatory and noninflammatory lesions; life quality (DLQI); porphyrins production; participants evaluation (TS)	Most common adverse effect: Skin irritation. The intervention group reported fewer adverse effects (skin desquamation and erythema) than the control group.
Mazzarello et al., 2018 [64]	RCT, 30 days	(PTA) 20 (1) 20 (2) 20	Mild to moderate; injury count	Propolis 20%, tea tree oil 3%, and *Aloe vera* 10%; cream; twice a day; cutaneous	(1) Erythromycin 3% (2) Placebo	Number of total lesions; number of inflammatory and noninflammatory lesions; acne severity (ASI); sebum production	NR
Orafidiya et al., 2004 [65]	RCT, 4 weeks	48/ (1) 12 (2) 12 (3) 12	NR; injury count	*Ocimum gratissimum* essential oil 2%, and *Aloe vera* (25%, 50%, and 100%); lotion; 0.25 cm^3^/twice a day; cutaneous	(1) Placebo (2) Negative control (*A. vera*) (3) Positive control (clindamycin)	Time necessary to reduce 50% the number of inflammatory lesions (days)	Intervention group: mild and tolerable adverse effects—96% of participants reported feeling a slight burning sensation on the skin.
Paranjpe and Kulkarni, 1995 [66]	RCT, 6 weeks	67/15	Moderate; injury count	4 ayurvedic formulations; pills; 500 mg/3 times per day; oral	Placebo	Number of inflammatory and noninflammatory lesions; participants evaluation (TR)	NR
Parveen et al., 2009 [67]	RCT, 2 months	20/10	NR; IGA	Unani formulation; cream; twice a day; cutaneous	Placebo	Acne severity (IGA)	NR

*Phytochemicals*							
Fabbrocini et al., 2011 [68]	Controlled clinical trials, 60 days	20/20	NR; GAGS	Resveratrol (0.01%, w/v); gel; once a day; cutaneous	Placebo	Acne severity (GAGS)	Without adverse effects
Jung et al., 2012 [69]	Uncontrolled trial, 8 weeks	30	Mild to moderate; injury count (inflammatory and noninflammatory lesions)	Polyphenon-60: catechin from green tea and is the representative green tea extract compound (20 mg/mL); lotion; twice a day; cutaneous	—	Number of inflammatory and noninflammatory lesions	NR
Yoon et al., 2013 [70]	RCT, 8 weeks	(Epigallocatechin-3-gallate 1%) 17/17 (Epigallocatechin-3-gallate 5%) 18/18	NR; modified Leeds system	Epigallocatechin-3-gallate 1% and 5%; solution; twice a day; cutaneous	Placebo	Number of inflammatory and noninflammatory lesions; acne severity (modified leeds system)	Intervention group (5%): erythema and skin irritation (*N* = 4). Intervention group (1%): without adverse effects.

ASI: Acne Severity Index; CADI: Cardiff Acne Disability Index; DLQI: Dermatology Life Quality Index; GAGS: Global Acne Grading System; GEA: Global Acne Severity Scale; IGA: Investigator's Global Assessment; KAGS: Korean Acne Grading System; NR: not eported; TR: treatment response; TS: treatment satisfaction.

## Data Availability

The data presented in this study are available upon request from the corresponding author.
